# Viscous fingering instabilities in spontaneously formed blisters of MoS_2_ multilayers†

**DOI:** 10.1039/d3na00563a

**Published:** 2023-10-30

**Authors:** Mukesh Pandey, Rajeev Ahuja, Rakesh Kumar

**Affiliations:** a Department of Physics, Indian Institute of Technology Ropar Rupnagar Punjab-140001 India rakesh@iitrpr.ac.in rajeev.ahuja@iitrpr.ac.in; b Condensed Matter Theory Group, Department of Physics and Astronomy, Uppsala University Box 516 Uppsala-75120 Sweden rajeev.ahuja@physics.uu.se

## Abstract

The viscous fingering in the Hele-Shaw cell can be suppressed by replacing the upper-bounding rigid plate with an elastic membrane. Recently, graphene multilayers while polymer-curing-induced blistering showed the dynamical evolution of viscous fingering patterns on a viscoelastic substrate due to their thickness-dependent elasticity. Under certain conditions, the elastic solid-based instability couples with the viscoelastic substrate-based instability. The mechanisms underlying such a coupling in the blisters of 2D materials and the dynamical evolution of the viscous fingering patterns underneath the blisters are yet to be addressed. Herein, we investigate the viscous fingering instabilities in spontaneously formed blisters of MoS_2_ multilayers, and provide thorough analytical and experimental insights for the elucidation of the dynamical evolution of the viscous fingering patterns and the coupled instabilities in the blisters. We also estimate the interfacial adhesion energy of the MoS_2_ flakes over a (poly)vinyl alcohol (PVA) substrate and the confinement pressure inside the MoS_2_ blisters using a conventional blister-test model. It is observed that the presence of instability gives rise to anomalies in the modeling of the blister test. The adhesion mechanical insights would be beneficial for fundamental research as well as practical applications of 2D material blisters in flexible optoelectronics.

## Abstract 2

A schematic representation of PVA-curing-induced blistering of MoS_2_ multilayers with and without the adsorption of tiny ice-water droplets (mist) over the PVA-coated Pyrex substrate prior to the mechanical exfoliation step.

## Graphical abstract

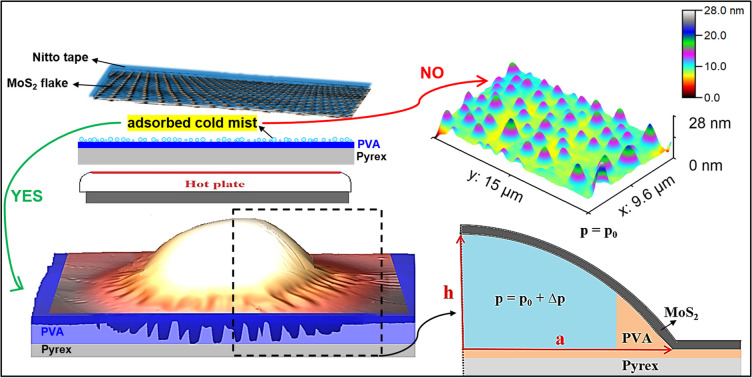

## Introduction

In recent years, the bending of 2D materials during the blister formation has been a subject of extensive focus due to their exceptional elasticity and the weak van der Waals (vdW) interlayer interactions.^1–4^ The 2D material blisters offer local strain sites with altered physical properties.^5–9^ Knowing the physics and chemistry underlying the intended^10–12^ or spontaneous formation^3,13–15^ of blisters is extremely important for the utilization or removal of 2D material blisters.^16,17^ It has also been demonstrated that the blisters of direct band gap semiconductors (*e.g.* transition metal dichalcogenides – TMDCs) act as ‘single photon emitters’.^18–21^ In addition, the blisters show remarkable photo-detection capability as the excitons have a longer lifetime against recombination due to the funneling effect.^22^ A blister test can be used to determine the intrinsic mechanical properties of a 2D material as well as its interfacial adhesion strength with a given substrate.^23–25^

The blistering of 2D materials over rigid solid substrates has been extensively investigated.^11,26^ However, less attention has been paid to the 2D material blister formation over soft polymeric substrates, possibly due to their considerable roughness and deformability. It's interesting to note that a hydrophilic polymer PVA acts as a potential substrate for mechanically exfoliated 2D materials, offering a larger deposition yield and monolayer area.^27,28^ It has been observed that the 2D material blister formation over the PVA substrate may lead to an unconventional phenomenon, *i.e.*, ‘viscous fingering’.^3^ This type of viscous fingering matches well with the Saffman-Taylor-like instability^29^ in which the length-scale of the polymeric fingers depends on the viscous and surface tension forces, elastic properties of the material, and the thickness of the confined viscoelastic film. On the contrary, the length-scale of surface undulations in the confined viscoelastic film developed due to the peeling of elastic/plastic sheets from the adhesive surface does not depend on the geometrical and material properties except the thickness of the film.^30–34^

The Saffman-Taylor-like instability, observed while debonding of a stiff plate from a viscous medium due to the displacement of a more viscous fluid by an injected less viscous fluid, gets suppressed when replacing the stiff plate with an elastic membrane.^35–37^ However, researchers have been unable to demonstrate a dynamic evolution of viscous fingering patterns using a single material. Recently, M. Pandey *et al.*^3^ showed for the first time that the graphene multilayer, having thickness-dependent elasticity, can show the evolution of viscous fingering patterns in a cold mist adsorption-assisted PVA-curing-induced blistering process. The adsorbed ice-water droplets over the PVA substrate locally manipulate the viscosity of the polymeric substrate surface, which alters its adhesive properties, and thereby makes the polymeric surface more deformable. The water vapor displaces the viscoelastic PVA at a raised temperature, and results in the formation of viscous fingering patterns underneath the 2D material blisters. The phase-transition of the confined matter inside a graphene blister induces hoop compression, whose suppression results in the formation of wrinkles around the perimeter of the blister or the tent at the center of the blister. The wrinkling is more prominent for blisters of thin 2D flakes (single to few layers) whereas the tent formation is more pronounced for thick 2D multilayers.^3^ Such kinds of elastic solid-based instabilities in the 2D material blisters arise as a result of (i) phase-transition of the confined matter,^3,38–40^ and (ii) interfacial sliding or contact failure.^41–43^ The hoop compression not only causes elastic solid-based instabilities but also has an impact on the viscoelastic substrate-based instabilities. M. Pandey *et al.*^3^ showed how the tent-like instability interacts with the viscous fingering instability underneath a multilayer graphene blister. It follows that it is quite possible for the viscous fingering instability at the interface to interact with the wrinkling instability developed around the perimeter of a blister of a relatively thin 2D elastic nanosheet. Such an investigation based on the 2D material blisters is currently lacking in the literature. However, through theoretical modeling, D. Pihler-Puzović *et al.* have demonstrated this interaction between the two types of instabilities in an elastic-walled circular Hele-Shaw cell.^44^

The onset of elastic solid or viscoelastic substrate-based instabilities and their mutual interaction depend on the strength of fluid–structure interactions, which is measured by the fluid–structure interaction parameter 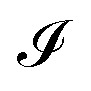, *i.e.*, the ratio of viscous stresses in the viscous fluid to the bending stiffness of the upper-bounding elastic membrane.^44,45^ In the cold mist adsorption-assisted PVA-curing-induced blistering of graphene multilayers, the water-vapor displaces the viscoelastic PVA in the in-plane direction inhomogeneously and simultaneously bends the elastic membrane in the out-of-plane direction, thereby forming the viscous fingering patterns underneath the blisters.^3^ At lower values of 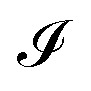, where the viscoelastic substrate exhibits smaller resistance to deformation than the upper-bounding elastic sheet, the viscous fingering instability predominates over the elastic solid-based instability. Chopin *et al.*^43^ demonstrated that the parameter *h*/*τ* (ratio of blister's height to flake thickness) regulates the shape profile of the blisters of a plastic sheet confining a liquid. M. Pandey *et al.*^3^ showed that the parameter *h*/*τ* not only regulates the solid-based instability or the shape profile of a multilayered 2D material blister but also the viscous fingering instability underneath the blisters over a viscoelastic substrate. For a single or few-layered 2D material blister, the solid-based instability occurs in the form of periodic wrinkles around the perimeter of the blister.^38^ Therefore, it is essential to comprehend the involved mechanism and the factors controlling the interaction between wrinkling and viscous fingering in the blisters. In the present work, we performed the polymer-curing-induced blistering of MoS_2_ multilayers under different synthesis and processing conditions to investigate the stability of the blisters and the interface based on the outcome of the blistering process. We utilize the 2D material blister-test model in the framework of ‘Föppl von Kármán theory of thin elastic plates’ and the two-phase fluid flow model in the framework of ‘simplified lubrication theory’ to elucidate the role of the interfacial velocity of the viscoelastic polymer while blistering and the interfacial adhesion strength of the 2D flakes with the substrate in the evolution of viscous fingering patterns underneath the blisters. We also provide intriguing insights into a scenario, where the viscous fingering instability couples with the wrinkling instability in the MoS_2_ blisters. We show that the presence of a solid- or substrate-based instability results in an anomaly in the modeling, where the parameter *h*/*τ* of the blisters attains unconventionally high values.

## Results and discussion

We analyzed our previous experimental findings on the PVA-curing-induced blistering of MLG flakes^3,9^ and observed that the conventional PVA-curing-induced blistering process results in 2D material blisters of lower *h*/*τ* with a stable and regular interface whereas the cold mist adsorption-assisted blistering process results in the blisters of larger *h*/*τ* with a complex interface. We further investigated the PVA-curing-induced blistering of MoS_2_ flakes under different synthesis and processing conditions to find the crucial parameter that is directly connected to the synthesis and processing conditions used in the experiment, whether it be the conventional or cold mist adsorption-assisted blistering process. M. Pandey *et al.*^9^ found that multilayered graphene blisters, formed spontaneously through the conventional PVA-curing-induced blistering process, follow the elastic plate model, which satisfies the condition *h*/*τ* ≲ 1.5, owing to significant bending rigidity of the multilayered flakes having minimal interlayer slippage. We find this observation to be also valid for multilayered MoS_2_ blisters formed through the same blistering process. But interestingly, when the PVA-coated Pyrex substrate is exposed briefly to a humid atmosphere at a lower temperature prior to the simultaneous curing and exfoliation step, the blisters of MoS_2_ multilayers form spontaneously with an exceptionally high *h*/*τ* ratio.^3^ The existing literature suggests that the parameter *h*/*τ* is larger (>2) for the blisters following the membrane profile.^46^ This hypothesis seems to be oversimplified because even if the condition *h*/*τ* ≲ 1.5 is no longer true, the blisters follow the nonlinear elastic plate profile, *i.e.*,1
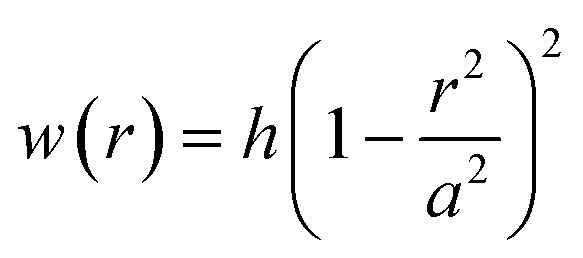
which is a fourth-order function of the radial distance *r*. The height profile of the blisters is fitted to a fourth-order polynomial function of radial distance *r* as, *w*(*r*) = intercept + *B*_1_*r* + *B*_2_*r*^2^ + *B*_3_*r*^3^ + *B*_4_*r*^4^, which gives central deflection or height *h* as the intercept and radius *a* as 
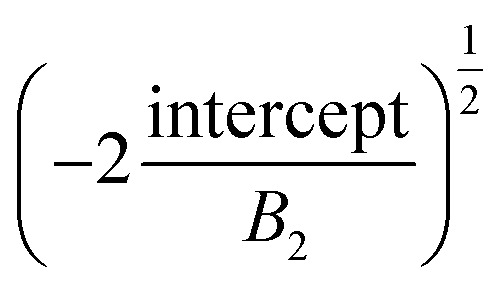
. We attribute the larger *h*/*τ* to the interlayer sliding due to weak vdW forces in between the layers of a multilayered 2D material. Therefore, we can reasonably assume that each layer bends independently while blistering such that the out-of-plane bending rigidity effectively scales linearly with the number of layers, *i.e.*, *B*_eff_ ∼ *N* (not *N*^3^).^1,47^ However, the level of interlayer sliding is more prominent (i) during the collapse of the blister due to the phase-transition of the confined matter, which gives rise to the tent-like shape of the blister, and also (ii) during the ice-water adsorption-assisted PVA-curing-induced blistering due to the ultralubricated interface resulting from the wetting.^3^ The interfacial adhesion energy density (*Γ*) of the PVA-supported MoS_2_ flakes consisting of *N* layers can be estimated using the equation:^9^2
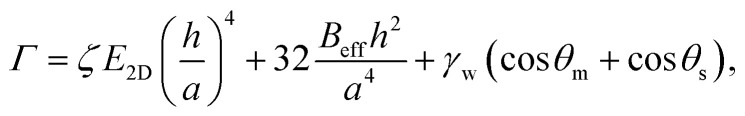
where *E*_2D_ = *Eτ* is the 2D elastic stiffness of the 2D flake, *τ* = *Nt* is the thickness of the 2D flake, *N* is the number of layers, *t* is the thickness of the monolayer, *E* is the in-plane elastic modulus of the 2D material, 
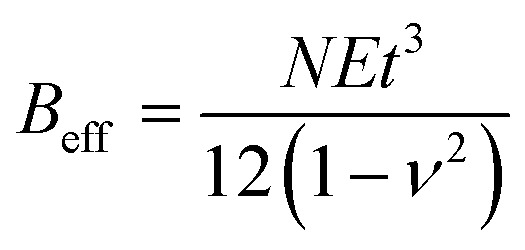
 is the effective bending stiffness, 
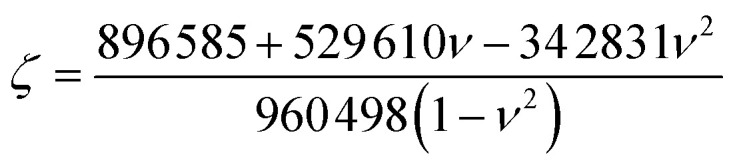
 is the constant prefactor, *ν* is Poisson's ratio, *γ*_*w*_ is the surface tension of water = 72 mN m^−1^, *θ*_m_ is the water contact angle with the 2D flake, and *θ*_s_ is the water contact angle with the substrate. For MoS_2_ blisters over the PVA substrate, we use the following parameters:^9,24,48,49^*E* = 270 GPa, *t* = 0.65 nm, *ν* = 0.29, *θ*_m_ = 69°, *θ*_s_ = 51°.

**Fig. 1:**
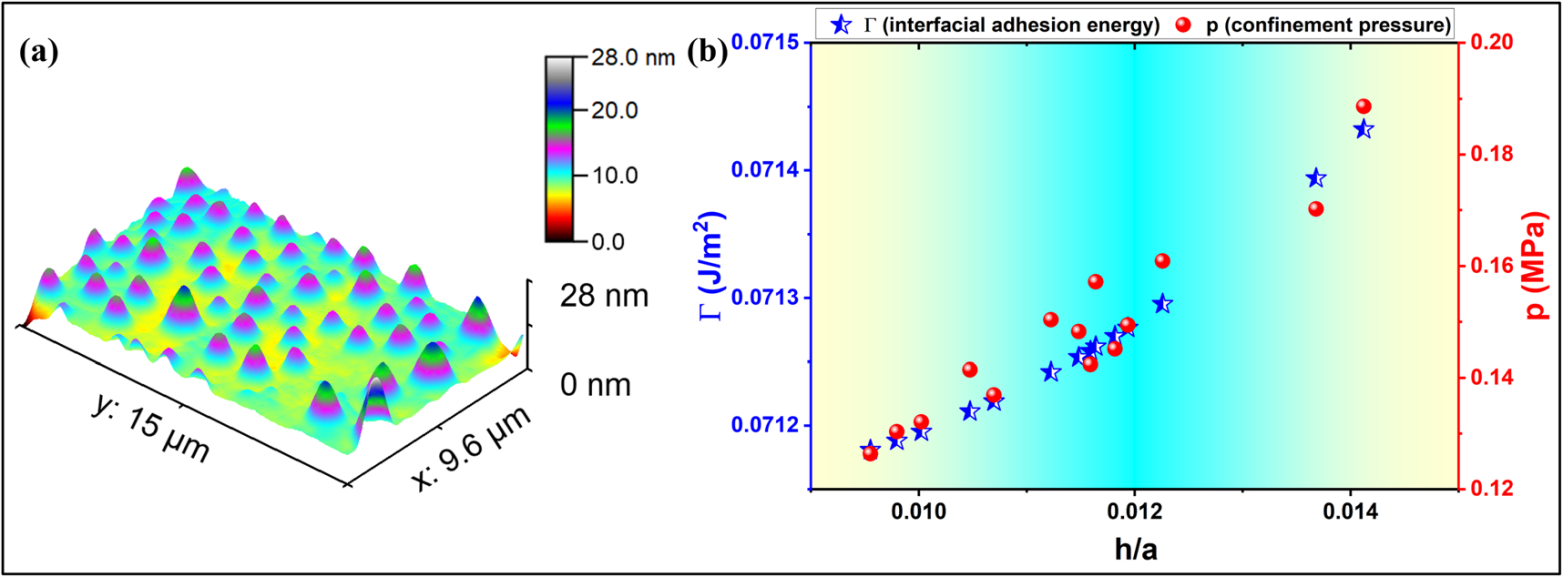
(a) Nanoblisters of multilayer MoS_2_, having the number of layers *N* = 39, formed spontaneously through the conventional PVA-curing-induced blistering process. (b) The interfacial adhesion energy and the net confinement pressure as a function of the blister aspect ratio for 14 different nano-blisters across the flake.

Hencky's model gives a relation between the confinement pressure (Δ*p*) and the topography of a blister,^46^ as3
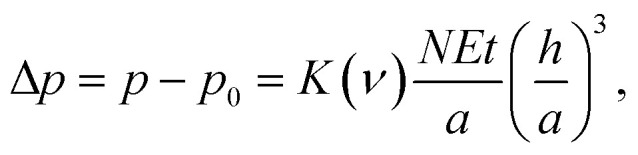
where *p*_0_ = 1 atm. pressure = 101 325 Pa, *p* is the net pressure inside the blister, and *K*(*ν*) is a constant prefactor; *K*(*ν* = 0.16) = 3.09 for graphene, and *K*(*ν* = 0.29) = 3.54 for MoS_2_.^24,48,50^ On employing the elastic plate model with the modified bending stiffness term for the MoS_2_ blisters formed through the conventional PVA-curing-induced blistering process, the interfacial adhesion energy (*Γ*) of the MoS_2_ multilayer (see Fig. 1) is estimated as ∼71.3 mJ m^−2^, and the net pressure inside the nanoblisters is ∼0.15 MPa.

**Fig. 2:**
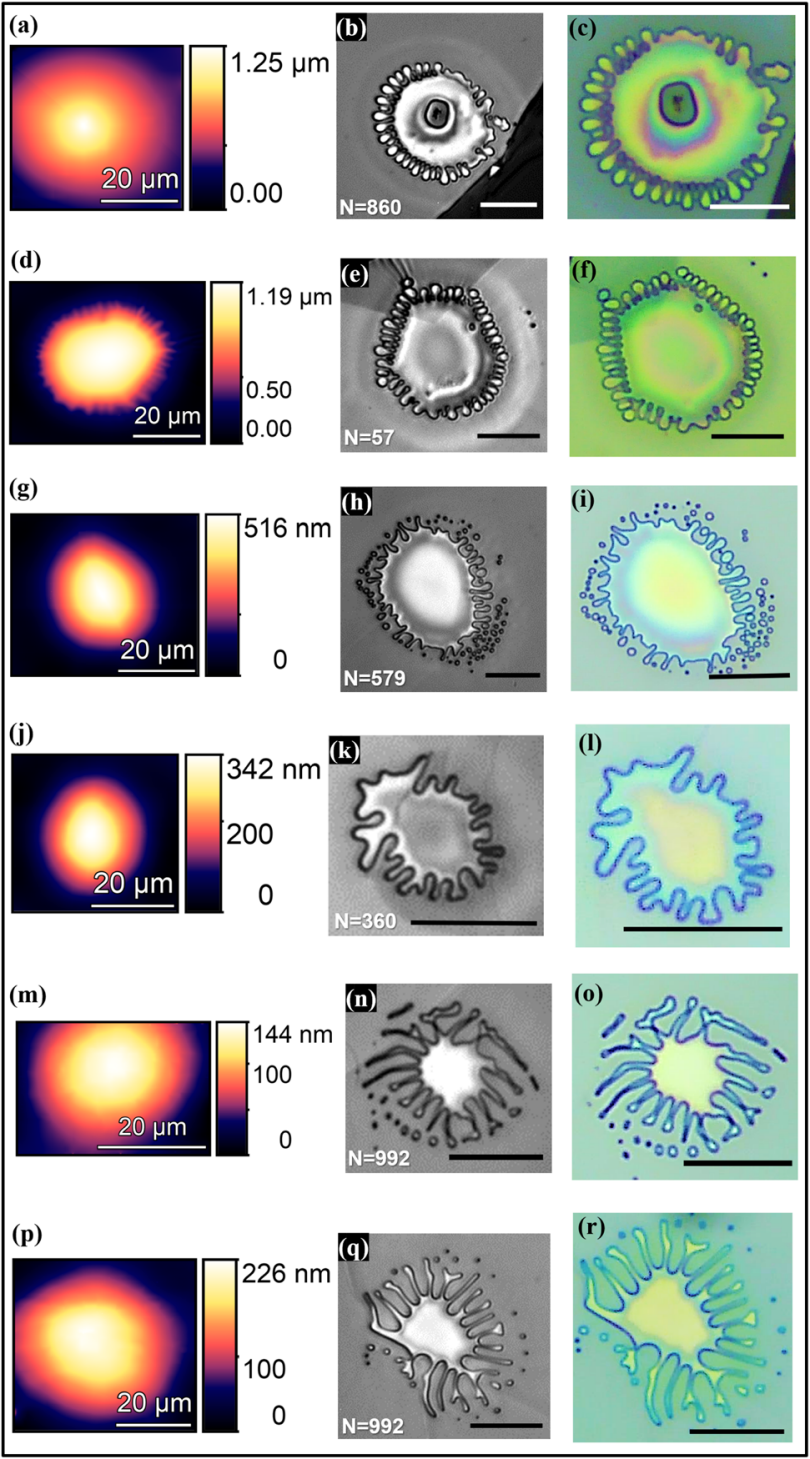
Blisters of MoS_2_ multilayers having viscous fingering instability underneath. The scale bar is 20 µm.

By employing the PVA-curing-induced blistering process after exposing the PVA surface to cold mist, we observed that the ratio of blister height to flake thickness (*h*/*τ*) dramatically increases, apart from observing the viscous fingering instability at the interface (see Fig. 2). It is clear from our observations that the elastic plate model that is applicable for *h*/*τ* ≲ 1.5 is violated in spontaneously formed blisters having at least one kind of instability, either the elastic solid-based instability (tenting or wrinkling), resulting from the phase transition of the confined matter, or the viscoelastic substrate-based instability (viscous fingering).^3,38^ To understand the underlying mechanisms, we employ the 2D material blistering model in the framework of the ‘Föppl von Kármán theory of thin elastic plates’ and the ‘simplified lubrication theory model’ for the two-phase fluid flow in an elastic-walled circular Hele-Shaw cell.^3,45^ The degree of instability in blistering is governed by the fluid–structure interaction parameter (dimensionless), *i.e.*,4
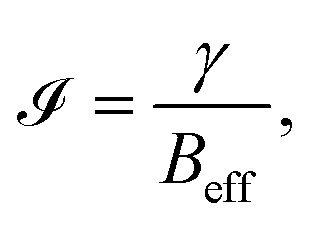
where *γ* is the viscous stiffness in the viscous fluid, and *B*_eff_ is the bending stiffness of the upper-bounding elastic membrane. The parameter 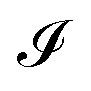 has an impact on the growth dynamics of viscous fingering patterns underneath the 2D material blisters. The parameter 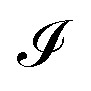 has two extreme limits: (i) 
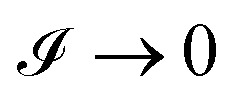
, which depicts the case where the viscoelastic substrate is easily deformable in comparison to the upper-bounding elastic membrane; this condition is responsible for the onset of viscous fingering instability; and (ii) 
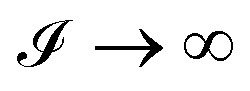
, which depicts the case where the upper-bounding elastic membrane is easily deformable in comparison to the viscoelastic substrate; this condition is responsible for the onset of solid-based instability. For a constant viscosity of the viscoelastic substrate, the parameter 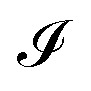 depends on the blister's length scale 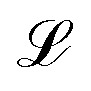 and the parameter *h*/*τ* at a unit time interval.^3^ The fluid–structure interaction parameter 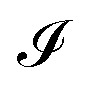 is related to the pressure for flux-driven flow *P*, as5
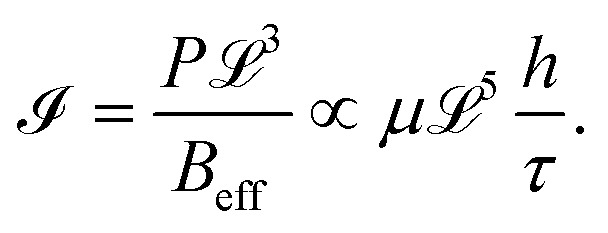


Assuming the flux-driven pressure to be of the order of the confinement pressure inside the 2D material blister, *i.e.*, *P* ∼Δ*p*. This yields an effective fluid-interaction parameter, as6
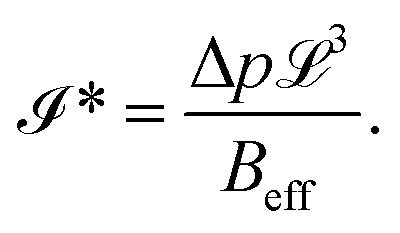


Assuming, 
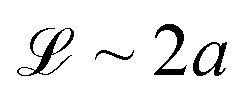
, and using eqn (3), we obtain7
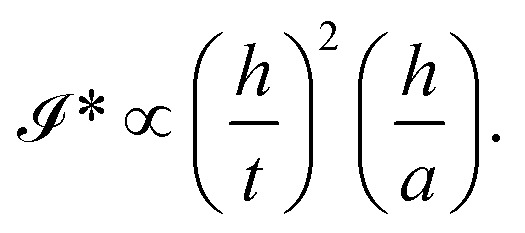


This approximation is valid for the case when the interlayer sliding is prominent due to weak vdW interactions, which is possible for an ultralubricated (frictionless) interface. Each layer of the multilayered flake attains the same bulging height *h* as that of the flake itself because each layer of the 2D material bends independently. Therefore, the effective thickness of the multilayered flake would be equal to the thickness of the single layer.^38^

**Fig. 3:**
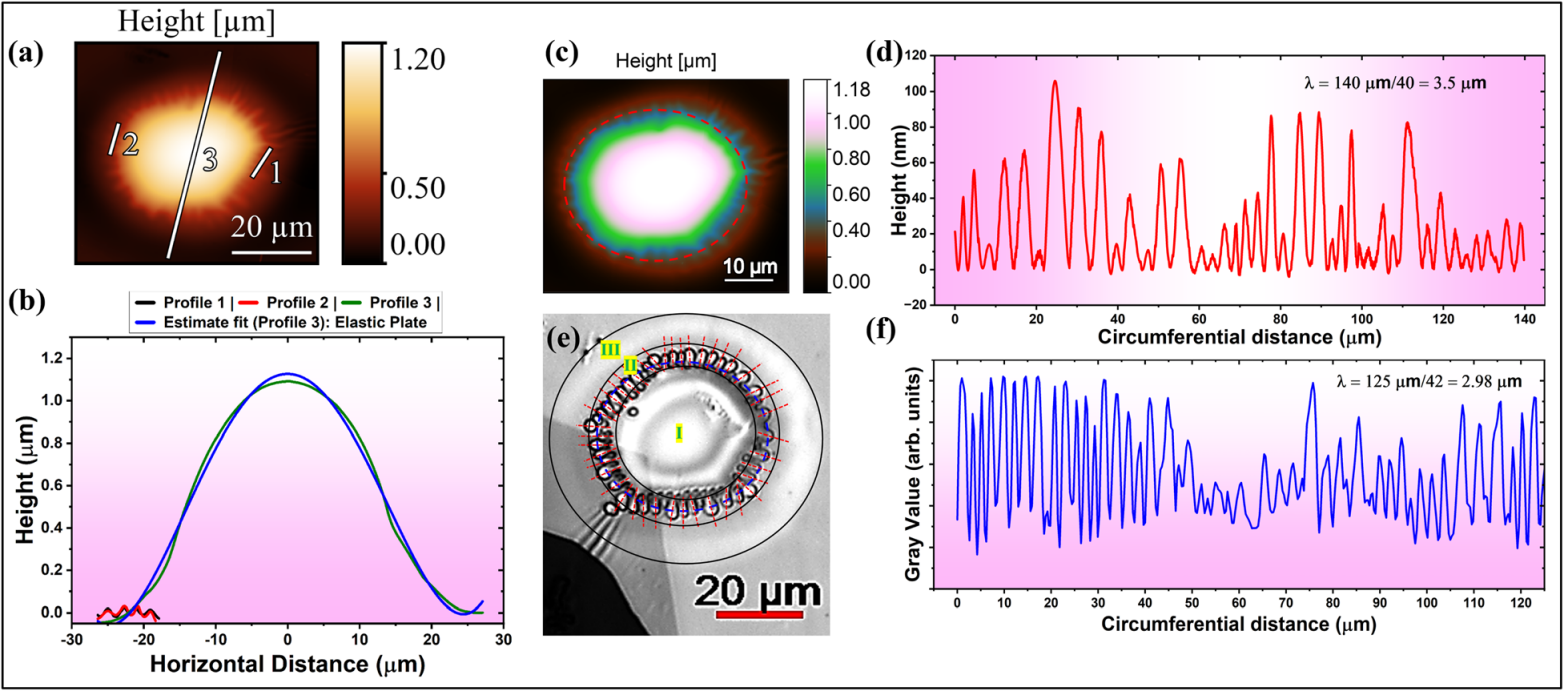
(a) AFM topographic 2D map of the blister with coupled instability; (b) AFM height profiles along the lines marked in (a). (c and d) AFM topographic 2D height image and the line profile across the periphery of the blister shown by a red dotted circle, respectively, and (e and f) optical image captured at the interface of the blister using interference reflection microscopy, depicting three zones of the blister, and the gray value plot profile across the periphery of the blister shown by a blue dotted circle, respectively.

**Fig. 4:**
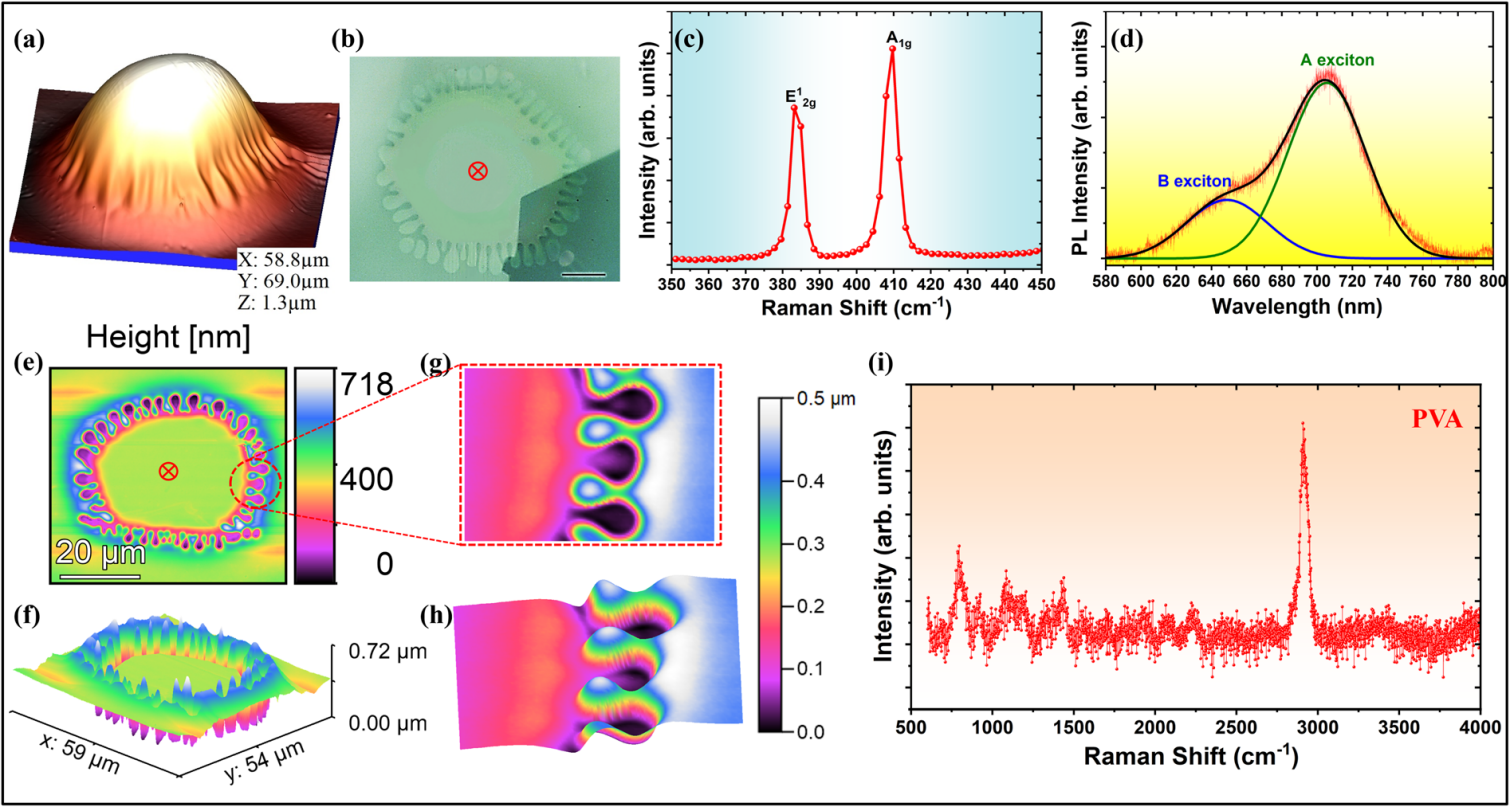
Multilayer MoS_2_ blister with coupled instability: (a) AFM topographic 3D image, (b) optical image (scale bar: 10 µm), (c and d) Raman and PL spectra acquired at a point (red-colored, as shown in (b)) located at the center of the blister, respectively, (e and f) AFM topographic 2D and 3D images of the viscous fingering pattern on the PVA surface, (g and h) top-view and side-view of the polymeric fingers, respectively, and (i) Raman spectrum acquired at the center of the pattern, indicating no degradation of the polymer.

We also observed the concurrence of wrinkling at the perimeter of blisters of thinner MoS_2_ flakes (*N* ≲ 100) and the viscous fingering at the interfaces (see Fig. 3). The 2D material blisters form at a raised temperature of ∼100 °C due to the trapping of water vapor resulting from the evaporation of water content (absorbed/adsorbed) of the PVA. The blister is of a circular shape at the raised temperature due to symmetrically distributed pressure across the blister walls, and it collapses at the edge due to condensation of the water vapor (gas) into the liquid phase as it is cooled down to room temperature.^3^ We observe three regions in a blister, as shown in Fig. 3(e), *viz.* (i) region I: the spherical or tent-like tip of the blister, (ii) region II: viscous fingering patterns at the periphery of the blister, and (iii) region III: the nearly deflated region of the initially circular blister due to phase transition-induced collapse. The tip height of the blister is almost 25 times larger than the average height of the wrinkles at the periphery of the blister (see Fig. 3(b)). On removing the bulged upper-bounding flake using a Nitto tape, the polymeric fingers formed at the periphery of a 2D material blister can be visualized (see Fig. 4(e)–(h)). It is clear from the topographic images (Fig. 4(g) and (h)) that the viscoelastic polymer PVA is radially displaced by the water vapor at the raised temperature. The polymeric fingers are captured by the wrinkles only at the edge of the blister, which depicts the interfacial nature of viscous fingering. The heat treatment (at ∼200 °C) of a PVA-supported 2D material blister in upside-down orientation can also reveal the type of viscous instability whether interfacial or bulk.^3^ Because of good thermal conductivity of the 2D material, the polymeric fingers at the periphery of the blister deform prior to the central region of the blister, depicting the interfacial nature of the viscous fingering.

Interestingly, we found that the wrinkle wavelength is nearly equal to the finger wavelength (see Fig. 3(d) and (f)). Therefore, the parameters governing the wrinkling in the thin MoS_2_ blister are directly related to the parameters for the onset of viscous fingering. Finding a critical criterion for such a situation to occur would indeed be worthwhile. According to the ‘local wavelength law’, the wrinkle wavelength is given by^38^8
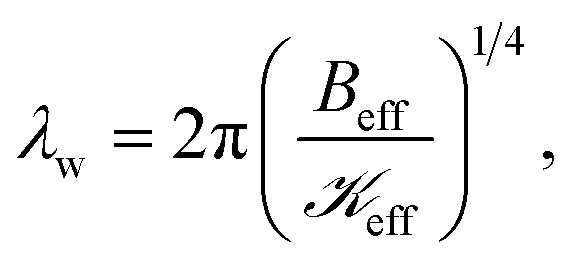
where 
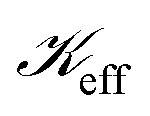
 is the ‘effective stiffness’, *i.e.*, the spring constant per unit area of a compliant substrate, having units of 
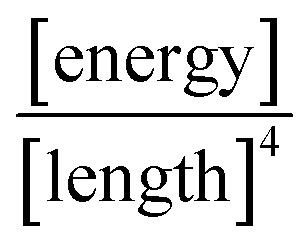
. The wavelength of the polymeric fingers, derived by Saffman and Taylor, is given by^44,45^9
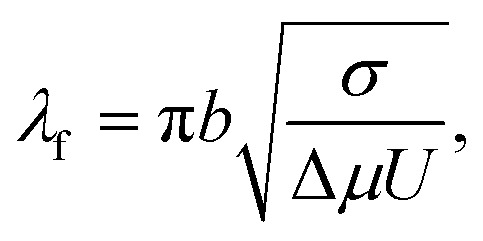
where *U* is the instantaneous radial velocity of the circular interface, *σ* is the surface tension at the water vapor to viscoelastic PVA interface, *b* is the undeformed initial thickness of the PVA film, and Δ*µ* = *µ* − *µ*_0_ is the viscosity difference between viscoelastic PVA and water vapor. Since the PVA is more viscous (*µ* ∼ 0.03 Poise) than the water vapor (*µ*_0_ ∼ 0.00013 Poise), the viscosity difference Δ*µ* ≈ *µ*.

On setting *λ*_w_ ≈ *λ*_f_, we obtain10
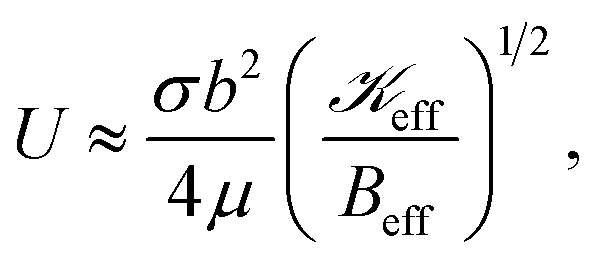
where 
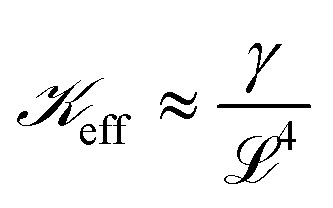
 manifests the energetic cost required to deform the viscoelastic substrate.^38^ However, in the case of blistering of a 2D elastic membrane over a 2D layered vdW crystal as a substrate, 
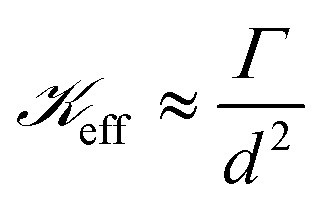
, *i.e.*, the ratio of interfacial adhesion energy density to the power 2 of the interlayer spacing in the 2D crystal. Near the glass-transition temperature of the PVA substrate, the surface tension (*σ*) and viscosity (*µ*) measurements of 4% w/w of partially hydrolyzed PVA solution (prepared in water) yield *σ* ∼ 38 mN m^−1^, and *µ* ∼ 3 mPa s, respectively. The thickness of the PVA film (*b*) is measured to be ∼100 nm. The interfacial velocity *U* can be rewritten as11
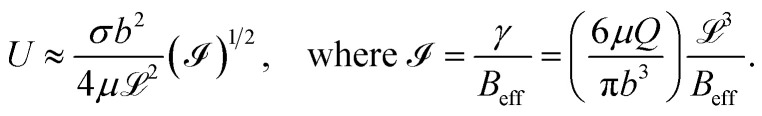


**Fig. 5:**
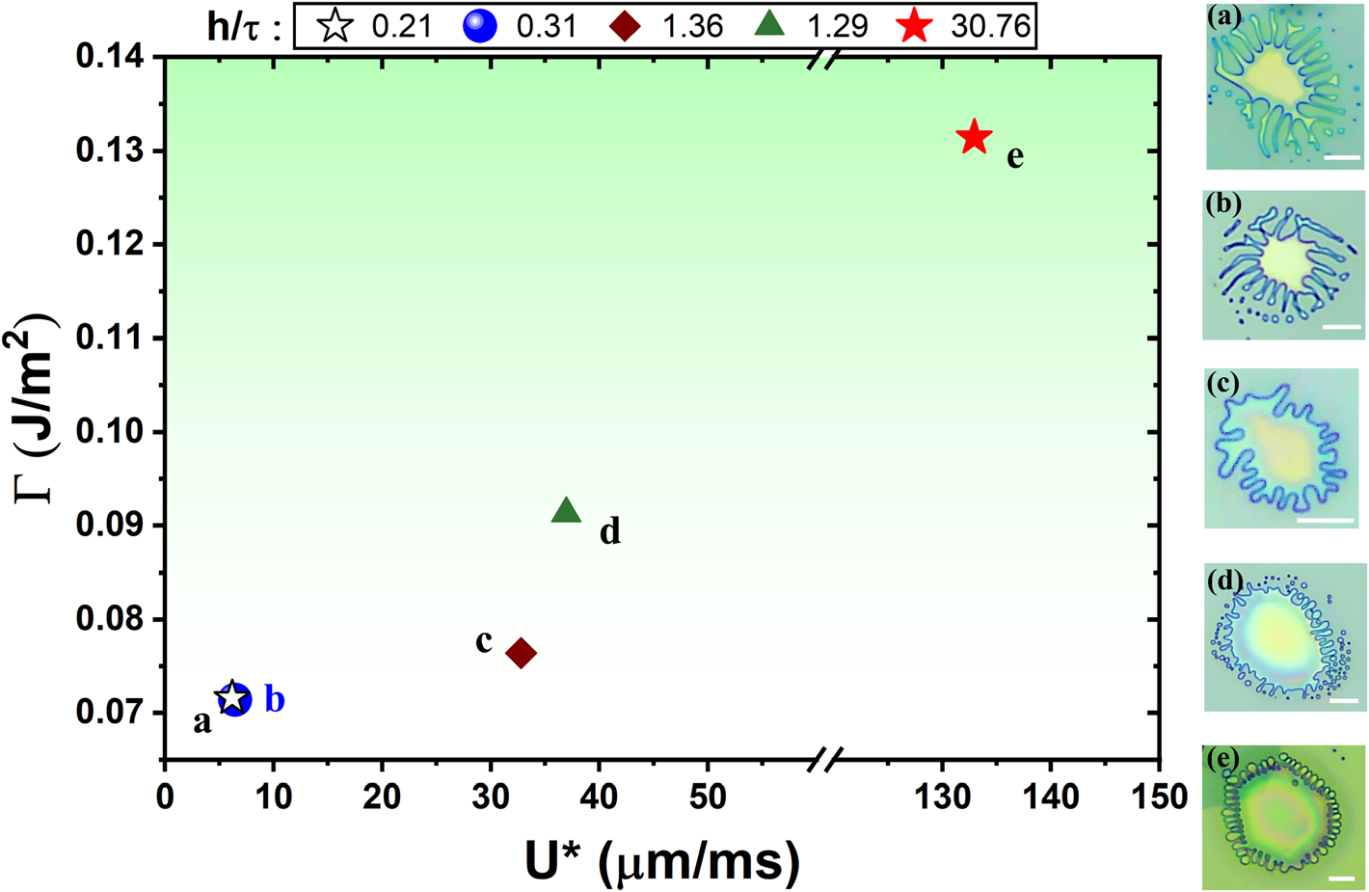
Graphical representation of circular blisters showing the dynamical evolution of interfacial viscous fingering instability with respect to the effective interfacial radial velocity (U*) and the interfacial adhesion energy (*Γ*). The scale bar is 10 µm.

For the volumetric flow rate 
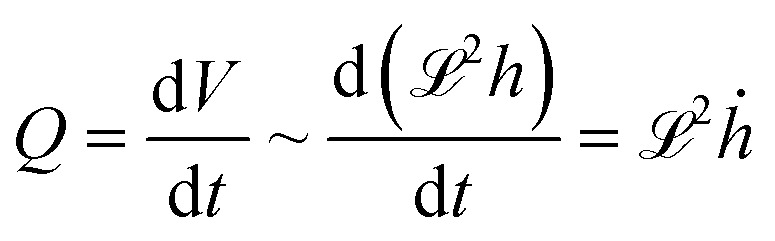
, and for a given system with fixed elastic and viscoelastic parameters, we obtain for all time:12
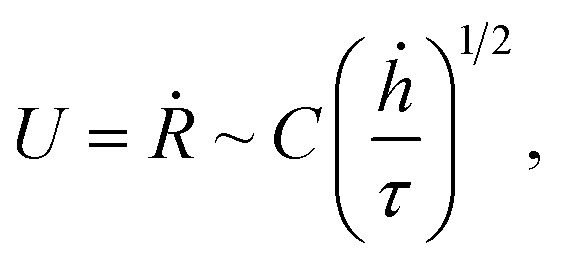
where *C* is a constant prefactor. Eqn (12) implies that a resonance between the in-plane velocity of the radially propagating viscoelastic fluid and the out-of-plane debonding velocity of the upper-bounding membrane leads to a condition where both the viscous fingering and the wrinkling instability in a blister interact with each other and occur concurrently. For the flux-driven pressure to be of the order of the confinement pressure, *i.e.*, 
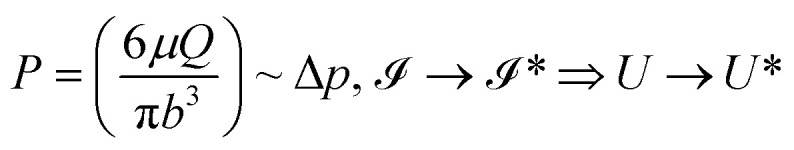
. It is to be noted that the atmospheric pressure outside the blister (*i.e.*, *p*_0_) remains constant, which has not been taken into account in the model to avoid complexities. This hypothesis effectively helped us in relating the growth dynamics of the viscous fingering patterns with the interfacial adhesion energy (*Γ*) and the effective interfacial in-plane velocity (U*) of the viscoelastic PVA being displaced by the water vapor while blistering (see Fig. 5). We observed that both the parameters (*Γ* and U*) simultaneously affect the growth dynamics of the fingering patterns.

The 2D material blisters with stable interfaces form spontaneously in the conventional PVA-curing-induced blistering process, where the stability of the interface is due to the higher viscosity and stronger viscous stresses of the PVA surface. The viscoelastic PVA remains typically static while PVA-curing-induced blistering under ambient conditions (see Fig. 1), however, under the cold-mist adsorption-assisted PVA-curing-induced blistering, the viscoelastic PVA radially propagates with an appreciable in-plane velocity and makes the interface unstable (see Fig. 5). The weaker the interfacial adhesion of the multilayered MoS_2_ flakes, the larger the polymeric finger length due to the smaller interfacial velocity of the viscoelastic PVA. The ice-water droplets locally reduce the viscosity of the PVA and make it quite deformable at raised temperatures (∼100 °C). The adsorbed water content or wetting results in the formation of blisters with exceptionally large values of the parameter *h*/*τ*, which indicates significant interlayer sliding during the blistering of the 2D multilayered flake. It should be noted that the blisters with a large *h*/*τ* ≫ 1.5 still follow the elastic plate profile. This observation suggests that the parameter *h*/*τ* is significantly influenced by the interlayer slippage as well as the interfacial adhesion strength.

## Conclusion

In summary, the synthesis and processing conditions, in addition to the interfacial debonding strength of the MoS_2_ multilayers, the confinement pressure inside the blisters, and the phase of the confined matter have crucial roles to play in the stability and dynamics of the blister system. The elastic properties of the 2D material, the viscoelastic properties of the polymeric substrate, and the physical properties of the confined fluid affect the blistering of the 2D multilayers. The adsorption of ice-water droplets on the hydrophilic PVA surface favors the interlayer sliding while blistering of the 2D multilayers at a raised temperature, which results in the exceptionally high values of the parameter *h*/*τ*, violating the non-linear elastic plate model. In the cold mist adsorption-assisted blistering of a 2D multilayer, the lower the interfacial radial velocity of the viscoelastic PVA, the larger the finger length. When the criterion of the resonance between the interfacial (in-plane) velocity of the polymer and the out-of-plane bending velocity of the 2D material is met, the solid-based wrinkling instability occurs concurrently with the viscous fingering instability. The mechanical insights of the blisters could potentially provide information about regulating the viscoelastic substrate-based instabilities in the blistering of the 2D layered vdW materials. The developed understanding might facilitate the next-generation applications of 2D materials and their blisters in flexible electronics, biomedical implants, single-photon detection, micro-/nanoelectromechanical sensing, *etc.*

## Experimental methods

### PVA-curing-induced blistering of MoS_2_ multilayers under different synthesis and processing conditions

The blisters of micromechanically exfoliated MoS_2_ multilayers are formed by a PVA-curing-induced blistering process both with and without the adsorption of tiny ice-water droplets (mist) over the PVA-coated Pyrex substrate prior to the mechanical exfoliation step. The steps in detail can be seen elsewhere.^3^

### Characterization

The identification of MoS_2_ blisters is carried out using interference reflection microscopy (IRM). The Raman and PL spectra have been acquired from the identified blisters using a HORIBA LabRAM HR Evolution system under ambient atmospheric conditions. The laser light output power is kept low (to prevent local heating) at ∼1 mW for the laser excitation wavelength of 532 nm using a 100× air objective lens (NA = 0.8) and a detector grating of 600 lines per mm in a confocal microscopy setup. The tapping-mode AFM measurements have been performed with a standard silicon cantilever using a Bruker MultiMode-8 AFM setup. The AFM data visualization and analysis have been carried out using the Gwyddion and WSxM software packages.

## Author contributions

Mukesh Pandey: conceptualization, methodology, data curation, investigation, formal analysis, visualization, validation, writing – original draft, writing – reviewing & editing. Rajeev Ahuja: writing – reviewing & editing. Rakesh Kumar: supervision, writing – reviewing & editing.

## Conflicts of interest

There are no conflicts to declare.

## Supplementary Material

NA-005-D3NA00563A-s001
