# Supplementary material for: Viscous fingering instabilities in spontaneously formed blisters of MoS2 multilayers
Source: Nanoscale Adv. 2023 Oct 30;5(23):6617–25. doi: 10.1039/d3na00563a (PMC10662142; doi:10.1039/d3na00563a)
Supplement: NA-005-D3NA00563A-s001 [file NA-005-D3NA00563A-s001.pdf]

Electronic Supplementary Material (ESI) for Nanoscale Advances.
This journal is © The Royal Society of Chemistry 2023

[Electronic Supplementary Information]

Viscous fingering instabilities in spontaneously formed blisters of MoS_2 multilayers

Mukesh Pandey, Rajeev Ahuja† & Rakesh Kumar*

Mukesh Pandey
T-GraMS Laboratory, Department of Physics, Indian Institute of Technology Ropar, Rupnagar, Punjab-140001, India.

Prof. Rajeev Ahuja
Condensed Matter Theory Group, Department of Physics and Astronomy, Uppsala University, Box 516, Uppsala-75120, Sweden;
Department of Physics, Indian Institute of Technology Ropar, Rupnagar, Punjab-140001, India.
† E-mail: rajeev.ahuja@physics.uu.se, rajeev.ahuja@iitrpr.ac.in

Dr. Rakesh Kumar
T-GraMS Laboratory, Department of Physics, Indian Institute of Technology Ropar, Rupnagar, Punjab-140001, India.
* Email: rakesh@iitrpr.ac.in

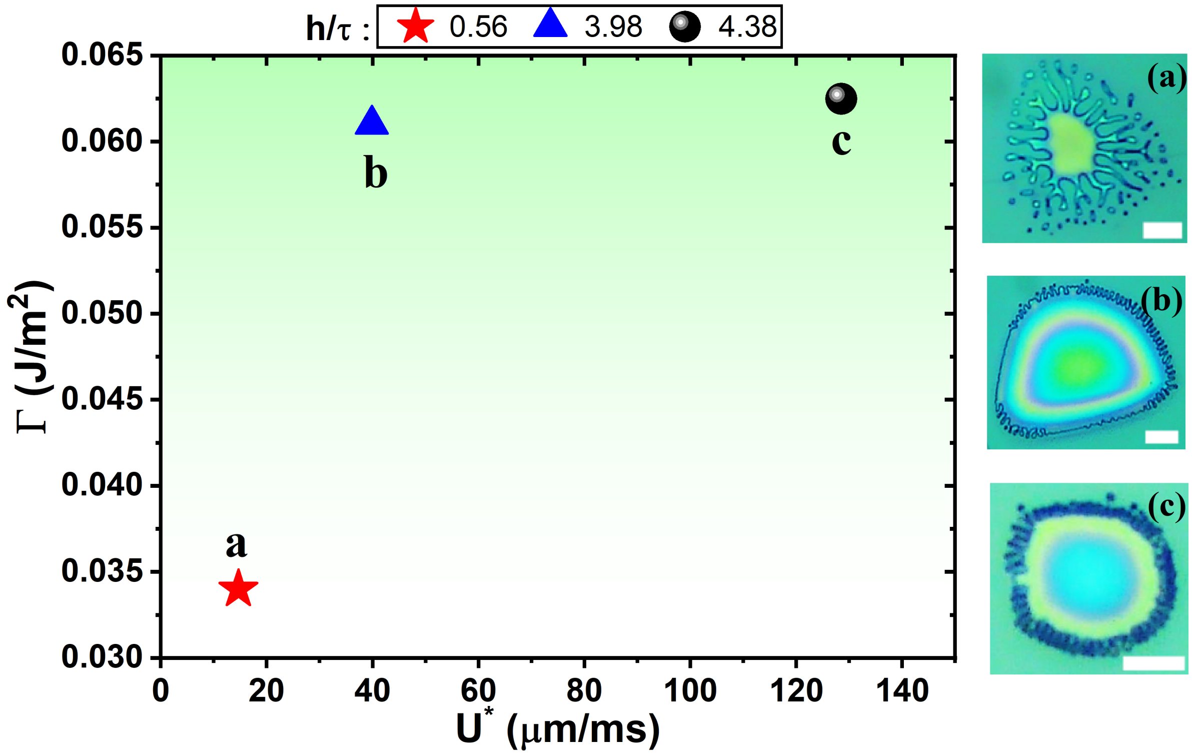

Figure S1 A graphical representation showing the dynamic evolution of viscous fingering patterns underneath multilayer graphene blisters as a function of the interfacial velocity of the radially propagating viscoelastic PVA and the interfacial adhesion of the upper-bounding membrane. The larger the interfacial adhesion of the membrane, the stronger the fluid (water vapor)-structure (MLG) interactions, which tends to suppress the viscous fingering instability at the interface[1].

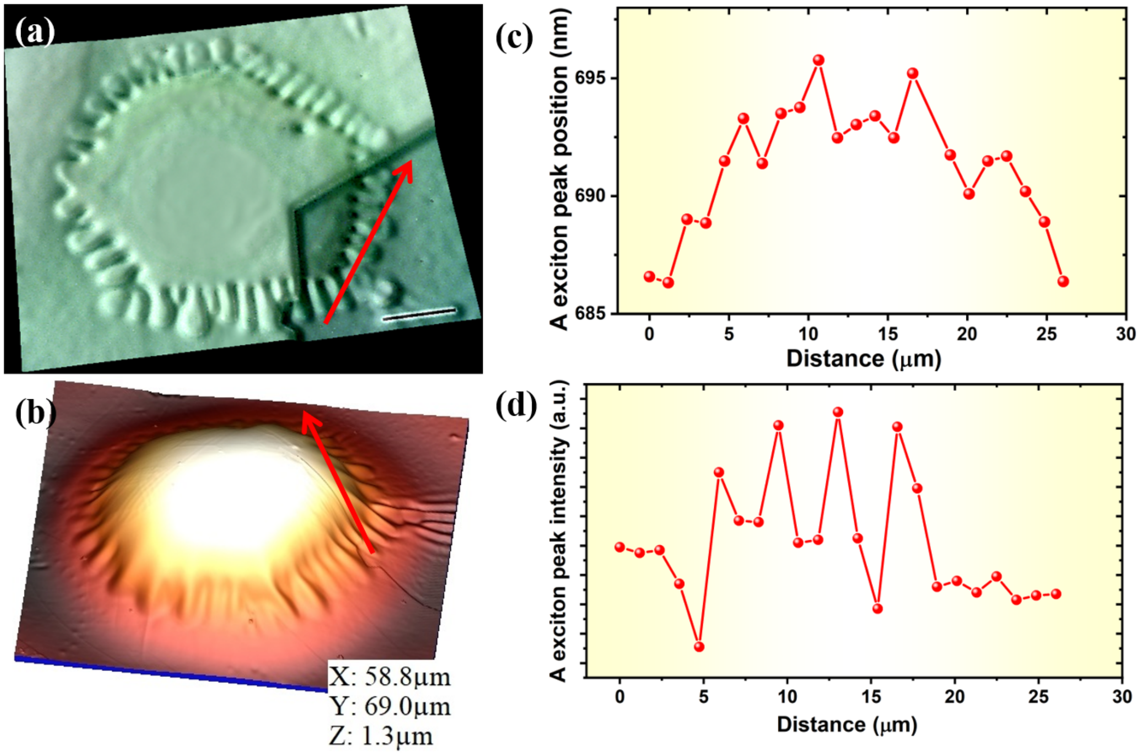

Figure S2 (a) Surface plot of the optical image; (b) AFM topographic 3D image of the blister; the arrow indicates the direction of the line map of the PL spectrum; (c, d) the line maps for the position and the PL intensity of the A-exciton peak, respectively. The optical band gap evolves across the curvature of the blister. The fluctuating line profile is because of the presence of wrinkles along the periphery of the blister.

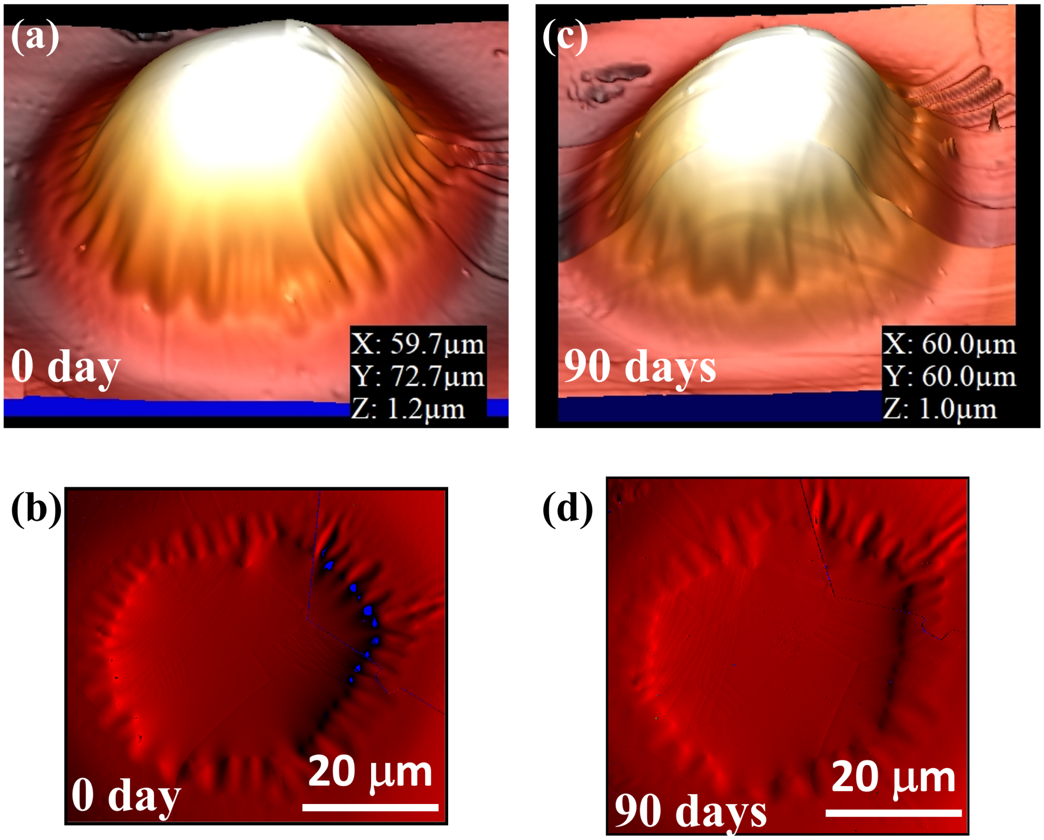

Figure S3 (a, c) The AFM 3D height images, and (b, d) the 2D amplitude images of a blister with coupled instability. The onset of wrinkling instability around the perimeter of a bubble of thin 2D flake is attributed to the phase transition of the confined water vapor during the cooling process[2]. The negligible time-lapse deflation of the polymer-curing-induced blisters at the ambient atmosphere indicates the liquid phase of the confined matter[3]. The blisters can sustain more than a period of 90 days. No new wrinkles were observed after the time-lapse, however, a few of the wrinkles vanish due to deflation.

References

[1] M. Pandey, R. Ahuja and R. Kumar, Hoop compression driven instabilities in spontaneously formed multilayer graphene blisters over a polymeric substrate, *Nanotechnology*, 2023, **34**, 175301.

[2] P. Ares, Y. B. Wang, C. R. Woods, J. Dougherty, L. Fumagalli, F. Guinea, B. Davidovitch and K. S. Novoselov, Van der Waals interaction affects wrinkle formation in two-dimensional materials, *Proceedings of the National Academy of Sciences*, 2021, **118**, e2025870118.

[3] D. A. Sanchez, Z. Dai, P. Wang, A. Cantu-Chavez, C. J. Brennan, R. Huang and N. Lu, Mechanics of spontaneously formed nanoblisters trapped by transferred 2D crystals, *Proceedings of the National Academy of Sciences*, 2018, **115**, 7884–7889.
